# The impact of malnutrition on childhood infections

**DOI:** 10.1097/QCO.0000000000000448

**Published:** 2018-04-26

**Authors:** Judd L. Walson, James A. Berkley

**Affiliations:** a The Childhood Acute Illness & Nutrition (CHAIN) Network, Nairobi, Kenya; b Departments of Global Health, Medicine, Paediatrics and Epidemiology, University of Washington, Seattle, Washington, USA; c KEMRI/Wellcome Trust Research Programme, Kilifi, Kenya; d Centre for Tropical Medicine & Global Health, Nuffield Department of Medicine, University of Oxford, Oxford, UK

**Keywords:** clinical trial, children, colonization, dysbiosis, environmental, growth, malnutrition, mortality, survival, susceptibility, undernutrition

## Abstract

**Purpose of review:**

Almost half of all childhood deaths worldwide occur in children with malnutrition, predominantly in sub-Saharan Africa and South Asia. This review summarizes the mechanisms by which malnutrition and serious infections interact with each other and with children's environments.

**Recent findings:**

It has become clear that whilst malnutrition results in increased incidence, severity and case fatality of common infections, risks continue beyond acute episodes resulting in significant postdischarge mortality. A well established concept of a ‘vicious-cycle’ between nutrition and infection has now evolving to encompass dysbiosis and pathogen colonization as precursors to infection; enteric dysfunction constituting malabsorption, dysregulation of nutrients and metabolism, inflammation and bacterial translocation. All of these interact with a child's diet and environment. Published trials aiming to break this cycle using antimicrobial prophylaxis or water, sanitation and hygiene interventions have not demonstrated public health benefit so far.

**Summary:**

As further trials are planned, key gaps in knowledge can be filled by applying new tools to re-examine old questions relating to immune competence during and after infection events and changes in nutritional status; and how to characterize overt and subclinical infection, intestinal permeability to bacteria and the role of antimicrobial resistance using specific biomarkers.

## INTRODUCTION

Worldwide, 5.6 million children die before their fifth birthday each year, with 80% of these deaths occurring in sub-Saharan Africa and Asia. Almost half of these deaths occur in children with malnutrition [1]. Strong epidemiological evidence suggests this is because of an elevated susceptibility to life-threatening infections amongst malnourished children. However, such studies do not disentangle the complex mechanisms underlying malnutrition, involving not only lack of nutrients, but also other risk factors such as exposure to pathogens, lack of access to healthcare and poverty. This review focusses on undernutrition among children in low and middle-income countries, with a focus on diarrhoea and pneumonia, the commonest childhood life-threatening infections worldwide. We discuss what is meant by the term ‘malnutrition’; how recent studies are informing our understanding of mechanisms linking anthropometric status and environment with susceptibility to life-threatening infections; and discuss implications and future research. 

**Box 1:**
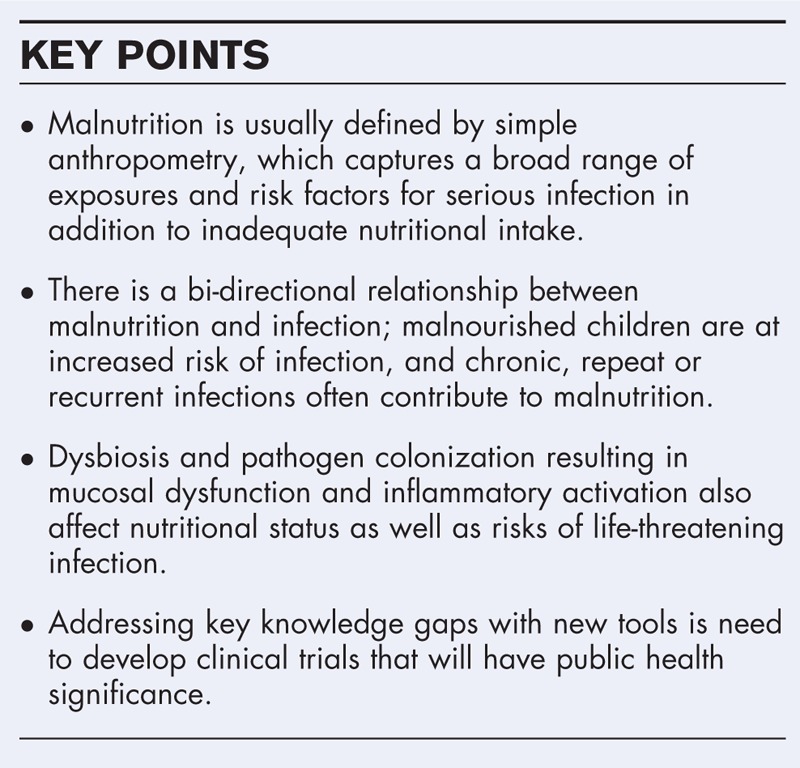
no caption available

## WHAT IS MALNUTRITION?

The World Health Organization (WHO) defines malnutrition as deficiency, excess or imbalance in a person's intake of energy and/or specific nutrients in relation to their requirements [2].

### Assessing malnutrition and risks of life-threatening infection

Energy and/or specific nutrient intake, requirements and expenditure are very rarely directly assessed. Instead, practice and research are based on anthropometric measures compared with a reference population. Wasting (thinness) is defined by weight-for-height/length (WHZ) among children under 5 years old, and BMI for age among 5–19-year olds. Stunting (linear growth impairment) is defined using height (or length)-for-age (HAZ).

Recently, there has been increased focus on the use of the mid-upper arm circumference (MUAC). MUAC is less affected by hydration status and generally it has better predictive value for subsequent mortality than WHZ [3,4]. However, cut offs to define malnutrition by MUAC based on its relationship with infectious disease or mortality outcomes had only been validated and used amongst children aged 6–59 months. Amongst infants (*n* = 2882) under 6 months old admitted to hospital in Kenya, MUAC was better at discriminating risk of subsequent inpatient death than WHZ [5]. A subset of these infants (*n* = 1405), were followed for 1 year after discharge; MUAC similarly had superior predictive value over WHZ. Similarly, amongst school-aged children and adolescents discharged from a rural hospital in Kenya (*n* = 1741) and amongst cohort of children over 5 years old with HIV infection in Uganda and Zimbabwe (*n* = 685), MUAC discriminated mortality risk at least as well as BMI-for-age [6]. This may result from MUAC directly measuring nutritional stores of protein (muscle) and fat whereas length is subject to significant measurement error among young infant.

Severe malnutrition can also be defined by the presence kwashiorkor, a syndrome characterized by nutritional oedema, often with skin depigmentation and sloughing, thinning of hair and inflammation. Why only some severely malnourished children develop kwashiorkor remains unknown [7]. Applications of new metabolomic, genomic and immunological techniques are addressing this question [8,9].

In addition to a diet low in energy or specific nutrients, a wide range of antenatal and postnatal environmental exposures, acute infection, chronic illness or psychosocial neglect may result in malnutrition [10]. It is clear from recent clinical trials enrolling children with severe malnutrition that they are also severely stunted (low height-for-age), suggesting chronic exposure to insult [11–13]. Hence, the commonly used term ‘severe acute malnutrition’ (SAM) has recently been challenged as a potentially misleading nomenclature with implications for successful intervention strategies [14^▪^]. We will use the term ‘malnutrition’ to mean low anthropometric values or kwashiorkor.

The interactions between episodic and chronic infections and malnutrition are complex and bi-directional. For example, children with malnutrition appear to be at substantially higher risk of diarrhoea, with both higher incidence and increased severity reported in malnourished children [15]. This risk appears to be correlated directly with degree of malnutrition as measured by anthropometry, with children with WAZ or HAZ 3 or less having a 37% increased risk of diarrhoea frequency and a 73% increase in average duration of diarrhoeal symptoms. At the same time, a meta-analysis assessing the impact of diarrhoea among several cohorts of children followed from birth until 24 months of age, demonstrated a 16% increase in stunting for every 5% increase in longitudinal incidence [16]. However, other studies have found mixed associations between frequent episodes of diarrhoea and long-term linear growth [17,18].

## OUTCOMES OF INFECTION

In addition to an increased frequency of infectious disease, children with malnutrition are at significantly higher risk of more severe disease and suffer significantly more acute and long-term morbidity and mortality when infected. Recently, a clearer separation between the acute condition and background risks has been made.

### Diarrhoea

Children with SAM are more likely to present to care with at least one integrated management of childhood illness (IMCI) danger sign and may be more likely to have a bacterial pathogen identified as a potential causative agent of their diarrhoea than nonmalnourished children [19^▪^]. In addition, as demonstrated in a study of 1146 children admitted to hospital with moderate-severe diarrhoea in Western Kenya (2005–2007), among children with severe acute malnutrition, risk of death following an episode of diarrhoea was four times higher than better nourished children [20]. The community-based Global Enteric Multicenter Study (GEMS) also enrolled 9439 children with moderate-to-severe diarrhoea and control children without diarrhoea in seven countries in Africa and Asia [21]. Diarrhoea case status was associated with stunting (chronic malnutrition leading to linear growth failure) as was postdiarrhoea mortality during 90 days, for which each *z* score unit of HAZ was associated with a reduction in the risk of death by 26–53% depending on age.

### Pneumonia

Similarly, malnutrition is not only associated with an increased risk of pneumonia episodes, but increased severity and case fatality. Development of an inpatient paediatric pneumonia mortality risk score (RISC) in Malawi (*n* = 16 475) [22,23], identified severe malnutrition as having similar predictive value to hypoxaemia and coma [21]. In Kenya, among 4187 children admitted to hospital with severe pneumonia, 25% were severely malnourished, again a strong risk factor for inpatient death alongside signs of disease severity [24^▪^]. A subset of children was followed after discharge from hospital; 37% of deaths occurred after discharge. Malnutrition, young age, HIV status and prolonged hospital admission were associated with postdischarge mortality, whilst pneumonia severity indicators were not, suggesting that an episode of severe pneumonia is a marker of background risk.

## MECHANISMS

**FIGURE 1:**
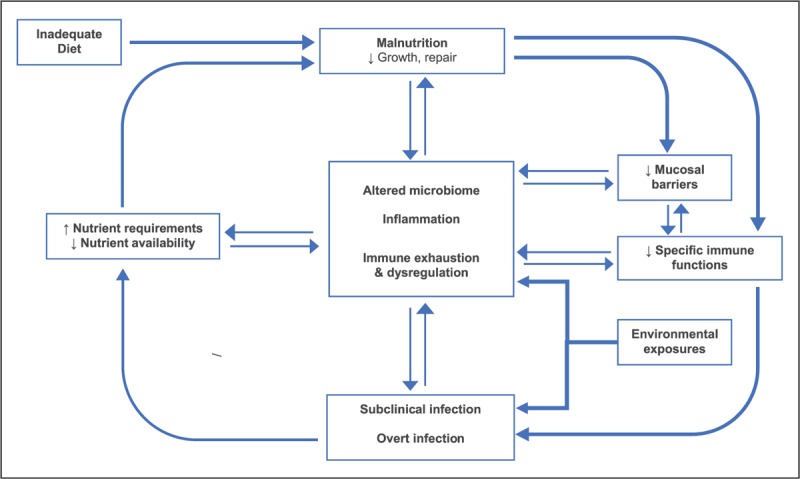
A growing understanding of a ‘vicious cycle’: interactions between malnutrition, infection and intestinal dysfunction.

A classic monograph by Scrimshaw in 1968, ‘Interactions of Nutrition and Infection,’ sets out a vicious cycle between nutrition and infection [25]. He proposed that malnutrition resulted in infections, infections resulted in malnutrition by anorexia, malabsorption, and diversion, loss, and increased requirements of nutrients. Currently, a more nuanced understanding is emerging of the roles of the environment, burden of exposure to pathogens because of crowding or poor water and sanitation, gut microbiota, chronic intestinal inflammation, mucosal barrier loss and immune function (Fig. 1).

### Dysbiosis and mucosal integrity

A key concept in understanding this relationship is that colonization of gut, respiratory and other mucosal surfaces, is a precursor to invasive infection. Malnutrition is typically accompanied by dysbiosis (change in the normal pattern of colonizing organisms) and disturbances in normal barrier functions.

The environment of the intestine plays a critical role as the main interface between the child and the nutrients and energy required to sustain growth. In addition to the critical function of modulating absorption and secretion, the enteric system is the predominant lymphoid tissue in the body. The surface of the gut functions as a major site of pathogen recognition and response, and is a critical barrier to pathogen translocation. Finally, the enteric system is a key site of hormonal modulation, regulating key functions related to metabolism and growth.

Although diarrhoea is a common manifestation of enteric infection and dysbiosis within the gut, many children experience significant intestinal dysfunction even in the absence of overt diarrheal disease. In many settings, chronic exposure to faecally contaminated environments may lead to an asymptomatic syndrome of poor absorption, local intestinal inflammation and increased translocation of bacterial products across the gut surface (environmental enteric dysfunction (EED) [26]. In many settings, markers associated with EED can be detected in as many as half of all children and these markers have been strongly associated with future linear growth failure [27,28].

EED can be identified by the presence of crypt atrophy and villous hyperplasia in the small intestine. In addition, a number of markers of intestinal permeability, absorption, inflammation and intestinal repair have been associated with the presence of EED [18,26,29]. These abnormalities lead to impaired absorptive capacity, local inflammation and disruption of tight junctions. This increases the potential for translocation of bacterial products and systemic immune activation. Although much attention has been paid to the local intestinal effects of EED on permeability, absorption and inflammation, the increased systemic inflammation and activation seen in malnourished children may be more important in explaining associations between malnutrition, gut dysfunction, long-term morbidity and mortality. In Malawi, both intestinal and systemic inflammation were associated with mortality risk in severely acute malnourished children and that this was not mediated by the presence of specific intestinal pathogens [30^▪^].

Similarly, malnutrition is associated with small intestinal histological abnormalities, including villous blunting, reduction in mucus-secreting goblet cells and inflammation [31]. It is not yet clear if this is EED or a separate disease. Previous metagenomic studies have suggested reduced microbial diversity in relation to a child's age and an increase in potent pathogenic Enterobacteriaceae in malnourished children [32]. In addition, several seminal studies have demonstrated growth failure in mice after receiving transplanted microbiota from malnourished children [33]. Increasing evidence suggests that the microbiome, both measured by diversity and taxa distribution, is a critical modulator of homeostasis within the gut, influencing absorption, immune function and hormonal regulation [34].

In addition to the general microbial milieu of the gut, specific pathogens have also been identified as potential mediators or drivers of malnutrition in some settings. In an urban community in Dhaka, Bangladesh, malnourished children (*n* = 486) and well nourished controls (*n* = 442) were investigated for a wide range of enteropathogens by Taqman Array Card. The presence of enteroaggregative *Escherichia coli,* heat-labile toxin producing *E. coli,* Shigella/enteroinvasive *E. coli, Campylobacter* spp., norovirus genogroup 1, and *Giardia* spp. were associated with malnutrition [35^▪^]. The number of different pathogens detected was inversely associated with subsequent growth, indicating clinically significant dysbiosis. This was further explored among 1684 children across eight sites in South Asia, Africa and Latin American by the Mal-ED study group [36^▪^]. Intestinal inflammation and growth were associated with the presence of enteroaggregative *E. coli*.

There are parallels at respiratory mucosal surfaces. In Ethiopia, nasopharyngeal carriage of *Streptococcus pneumonia* was assessed in 361 children at an outpatient clinic [37]. Overall, 44% were colonized by *S. pneumoniae* (not serotyped, 18% multidrug resistant) and colonization was associated with the number of siblings in the household and presence of malnutrition defined by weight-for-age, capturing aspects of both wasting and stunting: adjusted odds ratio 2.1 [95% confidence interval (CI) 1.2–3.4]. In Venezuela, amongst 1064 children living in rural areas of the Orinoco Delta, S. *pneumoniae* colonization was (nonsignificantly on multivariable analysis) more common among stunted children who were stunted [38]. However, a significant association had been previously shown in another population in Venezuela, with a 33% reduction in colonization per unit HAZ, and clear association between colonization and acute respiratory infection [39]. Pneumococcal colonization was not reported in relation to malnutrition in the multicentre PERCH study at nine sites in seven countries [40].

### Pathogens

Clinical outcomes in children with malnutrition might differ if they are infected with different organisms causing the same clinical syndrome (e.g. bacterial rather than viral cause), or if they present with the same pathogen but have an increased risk of antimicrobial resistance (AMR). The latter might occur with increased exposure to healthcare and antimicrobials or reduced pathogen clearance. In addition, children with malnutrition may simply respond differently to pathogen challenge, a range of abnormalities across multiple pathways in the innate and adaptive immune system have been described in these children [41,42]. Recent studies that standardize clinical conditions and their causative organisms are especially informative to our understanding.

Studies have demonstrated variable associations between malnutrition and bacteraemia risk. Some previous studies have suggested an increased likelihood of Gram negative bacteraemia in malnourished children [43]. However, the range of bacterial species is typically similar to those observed in nonmalnourished children in low-resource settings. One recent blood culture study from Tanzania reported a high prevalence of *Pseudomonas* spp. (36%)., *Enterobacter* spp. (16%), and *Staphylococus aureus* (15%), suggesting limited sensitivity to first line ampicillin with gentamicin. It is unclear if these were community-acquired (at admission) isolates or hospital-acquired (after admission) isolates. In Kenya, contrary to previous case series suggesting that coagulase-negative staphylococci (CONS, usually associated with invasive medical devices) may be important pathogens in severely malnourished children, there was no association between CONS being identified on blood culture and mortality or duration of hospitalization [44]. Further data is urgently needed on AMR in this context.

### Vaccine efficacy

Early studies of vaccine responses among malnourished children suggesting reduced efficacy of oral vaccines (polio, rotavirus), but no differences in antibody titres following parenterally given vaccines [45,46]. However, true efficacy or effectiveness against pathogen challenge remained uncertain. In a landmark South African case–control study, receipt of two or more doses of 13-valent pneumococcal conjugate vaccine was demonstrated to be as effective (90%) in preventing proven invasive pneumococcal disease among malnourished children as in well nourished children [47^▪▪^]. Thus, whilst altered vaccine efficacy may impact rotavirus disease in relation to malnutrition, it does not explain an increased susceptibility to common respiratory pathogens.

## CLINICAL TRIALS

Clinical trials have attempted to interrupt the cycle described above. In Kenya and Bangladesh, large-scale combined water, sanitation hygiene and nutrition interventions had minimal effects on diarrhoea or growth. In a multicentre trial in Kenya, long-term prophylaxis daily co-trimoxazole did not reduce postdischarge serious infections or improve growth during 1 year among severely malnourished children [12]. Trials of other antimicrobials to treat complicated severe malnutrition and/or prevent postdischarge mortality targeting dysbiosis and small intestinal bacterial overgrowth are underway [48]. Preventive trials using candidate probiotic organisms and prebiotic foods are also in progress as microbiota are better characterized [9,32]. Future trials may also involve systemic or gut-specific immunomodulation.

## FUTURE PERSPECTIVES

Despite a reduction in overall child mortality in the last 25 years, it is clear from recent modelling that most of Africa is highly unlikely to achieve the Sustainable Development Goal target of ending malnutrition by 2030 [49]. The Child Health and Mortality Prevention Surveillance (CHAMPS) project (https://champshealth.org) aims to determine causes of death in resource-poor populations through minimally invasive postmortem tissue sampling, which may better target therapy in life. The Childhood Acute Illness and Nutrition (CHAIN) Network (http://chainnetwork.org) aims to identify modifiable biomedical and socioeconomic risks to take forward in clinical trials.

## CONCLUSION

The malnutrition–environment–infection axis is complex and not easily addressed by individual interventions. Better understanding will come through applying new tools to re-examine longitudinal immune competence *ex vivo* in relation to infection events and changes in nutritional status, more specific biomarkers of infection, correlates of intestinal function and bacterial translocation, microbial populations and causes of disease.

## Acknowledgements

None.

### Financial support and sponsorship

The authors were supported by the Bill & Melinda Gates Foundation (grant number OPP1131320). J.A.B. is supported by the MRC/DfID/Wellcome Trust Global Health Trials Scheme (grant number MR/M007367/1).

### Conflicts of interest

There are no conflicts of interest.

## REFERENCES AND RECOMMENDED READING

Papers of particular interest, published within the annual period of review, have been highlighted as:

▪ of special interest
▪▪ of outstanding interest
